# The impact of acute kidney injury on fatality of ischemic stroke from a hospital-based population in Joinville, Brazil

**DOI:** 10.1590/2175-8239-JBN-2018-0215

**Published:** 2019-05-09

**Authors:** Helbert do Nascimento Lima, Tais Saibel, Gisele Colato, Norberto Luiz Cabral

**Affiliations:** 1 Universidade da Região de Joinville Departamento de Medicina JoinvilleSC Brazil Universidade da Região de Joinville, Departamento de Medicina, Joinville, SC, Brasil.

**Keywords:** Acute Kidney Injury, Kidney Function Tests, Stroke, Survival

## Abstract

**Introduction:**

The occurrence of acute kidney injury (AKI) after ischemic stroke has been associated to a worse prognosis. There is a lack of Brazilian studies evaluating this issue. This study aimed to describe the impact of AKI after a first-ever ischemic stroke in relation to fatality rate in 30 days.

**Methods:**

This was a retrospective hospital-based cohort. We included patients who had their first ischemic stroke between January to December 2015. AKI was defined by an increase of serum creatinine in relation to baseline value at admission ≥ 0.3 mg/dL or a rise in serum creatinine level by 1.5 times the baseline value at any point in the first week after admission. We performed a univariate and multivariate analysis to evaluate the presence of AKI with fatality in 30 days.

**Results:**

The final study population (n=214) had mean age of 66.46 ± 13.73 years, 48.1% were men, the mean NIHSS was 6.33 ± 6.27 and 20 (9.3%) presented AKI. Patients with AKI were older, had a higher score on the NIHSS, and had higher creatinine values on hospital discharge. The 30-day mortality was higher in the AKI subgroup compared to non-AKI (35% *vs*. 6.2%, *p* < 0.001). AKI was an independent predictor of fatality after an ischemic stroke but limited by severity of stroke (NIHSS).

**Conclusion:**

The presence of AKI is an important complication after ischemic stroke. Despite its impact on 30-day fatality, the predictive strength of AKI was limited by the severity of stroke.

## Introduction

Stroke is the third leading cause of death in developed countries and the leading cause of physical disability in people over 60 years old^1^. Despite a decrease in the mortality rate related to stroke in Brazil, the country still presents one of the highest risk of premature death after a stroke when compared to other countries in Latin America^2^. Among the possible factors related to fatality following a stroke, the presence of acute kidney injury has been increasingly considered as an important risk factor^3^^-^5; nevertheless, AKI has been little studied in Brazil.

Stroke was the main cause of death in all regions of Brazil among cardiovascular causes until 2011^2^. After this year, similar to developed countries, deaths due to ischemic heart diseases were the leading cardiovascular causes^2^. It is believed that part of this decrease in stroke mortality is associated with primary prevention measures adopted, such as smoking reduction and better control of arterial blood pressure^6^. However, mortality on the 30-day period after a stroke has a significant impact, with an estimated prevalence around 10%, as demonstrated by the Atherosclerosis Risk in Communities Cohort (ARIC) that studied approximately 14,000 individuals with stroke^7^.

Acute kidney injury (AKI) has been a frequent complication after an acute cerebrovascular event, with an overall prevalence around 11.6%^5^. More advanced age, presence of heart failure, diabetes, and ischemic heart disease have been associated with a higher risk of developing AKI after stroke^3^. The presence of AKI has been associated to higher mortality risk both in the short-term and long-term after an ischemic stroke^3^^,^^4^^,^^8^^,^^9^. However, part of the studies that demonstrated this association of AKI with worse prognosis after stroke did not consider the severity of the cerebrovascular event through standardized scales (*i.e.* National Institutes of Health Stroke Scale - NIHSS)^3^^,^^9^.

Considering the impact in the morbimortality of AKI after stroke and the lack of Brazilian studies exploring this relationship, the present study aimed to evaluate the prevalence of AKI in patients after the first-ever ischemic stroke and its impact in the 30-day mortality in a stroke public reference hospital for stroke.

## Methods

This was a retrospective hospital-based cohort study based on medical records and information from JOINVASC database from a population-based cohort study of patients with stroke in the city of Joinville, Brazil^6^. JOINVASC was designed to identified trends in Joinville, an industrial city with a population around 500,000 inhabitants. The JOINVASC methodology has been adopted in the stroke-steps modular program of the World Health Organization. The study was approved by the Ethics in Research Committees of the involved hospital.

The inclusion criteria were patients with a first episode of ischemic stroke from January 1 to December 31, 2015 and admitted in the São José Public Hospital (SJPH). SJPH is a reference institution for stroke cases, having a multidisciplinary care unit in stroke and medical residence in neurology. The exclusion criteria were patients younger than 18 years, subjects with incomplete data, and those in chronic dialysis treatment.

The diagnosis of ischemic stroke was established by a neurologist based on the presence of focal or global signs of cerebral dysfunction lasting more than 24 hours and with no apparent non-vascular cause. In addition, the diagnosis was confirmed by compatible findings of computed tomography or magnetic resonance imaging within 24 to 72 hours after admission, as defined by the World Health Organization criteria^10^. Subsequently, during admission, an experienced nurse collected information about comorbidities, other preexisting risk factors, and sociodemographic data according to self-reported previous history. Values of systolic and diastolic blood pressure were measured on the emergency room during admission, and routine laboratory exams were performed. AKI was defined by an increase of the serum creatinine in relation to baseline value at admission ≥ 0.3 mg/dL or a rise in the serum creatinine level by 1.5 times or more within the last 7 days after admission, as defined by Kidney Disease Improving Global Outcomes (KIDGO) and considered in other similar studies^4^^,^^5^^,^^11^^,^^12^. Criteria considering urine output were not used in this study once urine output was not consistently recorded in all patients.

## Statistical analysis

The qualitative variables are presented as the absolute numbers and their percentages and quantitative variables by their mean and standard deviation. The differences between the frequencies of the qualitative variables were analyzed using the chi-square test and quantitative variables by Student’s t-test or the Mann-Whitney test, according to data distribution. AKI defined as KDIGO stage 1 or greater was used in the models. We performed a univariate analysis of the variables with clinical relevance for the outcome, death in 30 days. Then, we performed two multivariate analysis (with or without NIHSS score) through logistic regression with the variables that showed a *p* value ≤ 0.100 in the univariate analysis. In the multivariate analysis, statistical significance was considered if *p* value < 0.05. Associations are presented as odds ratio and corresponding 95% confidence intervals (95% CI). A Kaplan-Meier survival curve of 30-day mortality was generated considering the presence of AKI. The analyzes were performed using SPSS-23 software.

## Results

From January to December 2015, a total of 317 patients were admitted in the SJPH with a first episode of ischemic stroke. One hundred and three patients were excluded: 3 patients due to being on chronic hemodialysis and 100 patients for incomplete data. Fifty two percent of the excluded sample was men, with mean age of 69.26 years, and a mean NIHSS of 6.23.

The final population study (n=214) had mean age of 66.46 ± 13.73 years, 48.1% were men, the mean NIHSS was 6.33 ± 6.27, and 20 people (9.3%) presented AKI. The group with AKI was older and had higher creatinine values on discharge. Patients with AKI presented higher 30-day mortality compared to patients without AKI (35.0% versus 6.2%, *p* < 0.001). The difference between the mean time to death was approximately 6 days less for the group with AKI in relation to those without AKI. Eighty-four percent of patients that died in 30 days were older than 65 years and the 84% had an NIHSS score higher than 14. The other characteristics of the study population as well as stratified by AKI presence or absence are presented in Table 1.

**Table 1:** Baseline characteristics of ischemic stroke in the total sample and by presence or absence of acute kidney injury (AKI)

		Total Sample (n = 194)		With AKI (n = 214)		Without AKI (n = 20)		*p* value
Age (yr; mean [SD])		66.16	13.66	65.58	13.45	75.00	13.77	0.006
Female Gender (n [%])		111	51.9	99	51.0	12	60.0	0.597
Race, white (n [%])		193	90.2	175	90.2	18	90.0	1.000
BMI (kg/m^2^; mean [SD])		16.39	4.77	26.42	4.79	25.90	4.39	0.955
Previous Comorbidities (n [%])								
Hypertension		153	71.5	138	71.1	15	75.0	0.917
Diabetes		71	33.2	64	33.0	7	35.0	1.000
Cigarette Smoking		106	49.5	99	51.0	7	35.0	0.258
Ischemic Heart Disease		20	9.3	19	9.8	1	5.0	0.701
NIHSS Score (n [%])								0.006
NIHSS ≤ 4		117	54.7	110	56.7	7	35.0	
NIH 5 to 14		70	32.7	64	33.0	6	30.0	
NIH > 14		27	12.6	20	10.3	7	35.0	
SBP on admission (mmHg; mean [SD])		154.92	28.81	154.71	27.28	163.55	36.52	0.450
DBP on admission (mmHg; mean [SD])		88.47	16.91	88.05	15.93	90.75	22.98	0.614
Subtypes of Ischemic Stroke (n [%])								0.400
Atherothrombotic		28	13.1	25	12.9	3	15.0	
Cardioembolic		29	13.6	27	13.9	2	10.0	
Lacunar		73	34.1	69	35.6	4	20.0	
Other		84	39.3	73	37.6	11	55.0	
Laboratory Values (mg/dL; mean [SD])								
Total Cholesterol		185.29	39.52	187.70	45.29	187.50	50.75	0.699
HDL Cholesterol		41.29	11.83	41.13	12.45	47.38	13.11	0.053
LDL Cholesterol		112.84	31.93	114.53	37.26	116.75	31.97	0.797
Triglycerides		154.50	84.00	157.38	87.68	154.44	98.55	0.624
Glucose		123.41	48.92	125.73	51.04	142.80	72.54	0.535
Creatinine on admission		0.91	0.37	0.91	0.37	0.88	0.38	0.659
Creatinine on discharge		0.88	0.38	0.86	0.35	1.12	0.57	0.013
Staging of AKI by KDIGO								
Stage 1		5	25.0					
Stage 2		13	65.0					
Stage 3		2	10,0					
Length of Stay (days; mean [SD])		15.70	11.76	15.14	11.04	21.10	16.68	0.120
Time to death (days; mean [SD])		28.21	6.35	28.71	5.46	23.45	11.06	< 0.001
Death in 30-days (n [%])		19	8.9	12	6.2	7	35.0	0.001

BMI= body mass index; NIHSS= National Institutes of Health Stroke Scale (values from 0 [best score] to 36 [worst score]); SBP= systolic blood pressure; DBP=diastolic blood pressure; AKI=acute kidney injury. KIDGO= Kidney Disease Improving Global Outcomes.

**Figure 1:**
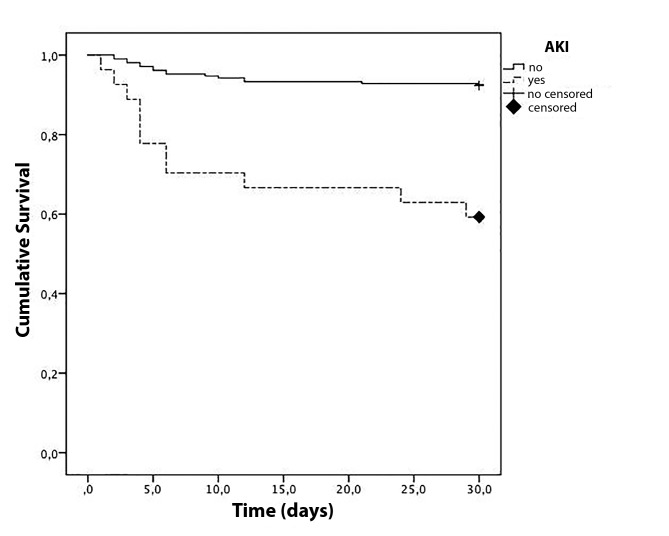
Survival curve for patients after ischemic stroke with or without acute kidney injury (AKI).

From the Kaplan-Meier analysis, the mean time for the 30-day mortality was 23.45±2.41 days (95% CI: 18.72-28.17) for the group with AKI and 28.71±0.41 days (95% CI: 27.90-29.51) for the group without AKI (*p* < 0.001; Figure 1).

In the univariate analysis, the predictors related to mortality in 30 days after an ischemic stroke were: presence of acute kidney injury, age, NIHSS score, and previous history of ischemic heart disease (Table 2).

**Table 2:** Univariate Analysis to predict death in 30 days after ischemic stroke

Variable	OR	95% CI	*p* value
AKI	7.74	2.19-27.33	0.001
Female Gender	0.93	0.33-2.58	0,884
NIHSS score	1.26	1.14-139	< 0.001
Age	1.05	1.00-1.09	0.044
BMI	0.94	0.84-1.06	0.326
Hypertension	0.77	0.24-2.44	0.654
Diabetes	0.74	0.24-2.29	0.603
Cigarette Smoking	1.03	0.37-2.85	0.961
Dyslipidemia	0.68	0.24-1.90	0.463
Ischemic Heart Disease	3.96	1.09-14.38	0.037

AKI=acute kidney injury; NIHSS=National Institutes of Health Stroke Scale; BMI=body mass index.

In the multivariate analysis, presence of AKI and previous ischemic heart disease were a predictor of a higher fatality rate only when NIHSS was removed from the regression model. Higher stroke severity score and age were predictors of a higher fatality rate in both multivariate models (Table 3).

**Table 3:** Multivariate analysis to predict death in 30 days after ischemic stroke

Model 1			
	OR	95% CI	*p* value
AKI	4.24	0.78-23.09	0.095
Ischemic Heart Disease	3.75	0.72-19.64	0.118
Age	1.04	0.99-1.10	0.142
NIHSS score	1.24	1.11-1.38	< 0.001
**Model 2**			
	**OR**	**95% CI**	***p* value**
AKI	8.65	2.19-34.26	0.002
Ischemic Heart Disease	5.09	1.22-21.30	0.026
Age	1.03	0.98-1.08	0.271

AKI=acute kidney injury; NIHSS=National Institutes of Health Stroke Scale.

## Discussion

Based on our literature review up to October 2018, this is the first Brazilian study that evaluated the impact of AKI on the short-term prognosis of patients with first-ever ischemic stroke. Our study demonstrated that the presence of AKI is a relevant complication after ischemic stroke and an independent predictor of fatality within 30 days when stroke severity is not considered.

AKI has been a common problem for patients after stroke^3^^,^^4^. According to a meta-analysis, which included 12 studies with more than 5 million stroke patients, the prevalence of AKI was 11.6% (95% CI: 10.6-12.7%)^5^. Our study found a lower prevalence even considering the same definition criteria for AKI from that meta-analysis. The presence of AKI has been associated with more advanced age, presence of previous heart failure, and atrial fibrillation, as well as more severe cases of stroke^3^^,^^13^. In contrast with other studies that showed a higher prevalence of AKI^3^^,^^9^, our study population did not include patients with previous cerebrovascular events, which might justify our lower prevalence of AKI.

Different from other studies, in which the presence of AKI was independently associated to a higher 30-day mortality after ischemic stroke^4^^,^^9^^,^^14^, our study did not find such an association when considering stroke severity. Despite our fatality rate being similar to other studies^5^, AKI lost predictive strength when considering stroke severity through NIHSS. The NIHSS score has been established as a very important predictor of short and long-term mortality after stroke^15^. There is a graded relationship between an increasing NIHSS score and higher fatality in 30 days after stroke^15^. Such an association has already been demonstrated in other studies. The score has also been related to an increased risk for a worse outcome after a stroke^16^^,^^17^. An NIHSS score higher than 15 is associated to a high risk of death in relation to a score below 6^16^^,^^18^. Similarly, older age of patients with ischemic stroke at admission has been well established as a predictor of a higher fatality rate in 30 days^19^^,^^20^. Older people are more likely to present a bad prognosis after a stroke due to previous disease and stroke severity than younger people^21^. In our study, the majority of patients that died presented a NIHSS score above 14 and were above the median age of 65. We believe that the presence of AKI characterized just by the initial definition criteria from KDIGO^22^ might not have been enough to affect the strength of NIHSS and age in our study. Besides that, cases of increased creatinine could have been falsely attributed to AKI, as some other factors might acutely increase creatinine values without clear presence of AKI (e.g. hyperglycemia and dietary intake)^12^.

AKI requiring dialysis is an important cause of death on the short and long term, even after a recovery of kidney function^23^^,^^24^. This higher mortality risk is partly associated to the traditional cardiovascular risk factors commonly found in patients with AKI. The higher risk might also be partly associated to production of inflammatory cytokines involved in the regenerative process of the tubular epithelial cells^23^^,^^24^. None of the patients in our study needed acute hemodialysis. Patel *et al.* reported trends for a decrease of fatality rate in patients with AKI after ischemic stroke in the last few years^25^; however, the number of those with AKI requiring hemodialysis has increased^26^. Part of the mortality burden associated to hemodialysis in AKI situations is due to the complications associated with the use of central venous catheters (e.g. sepsis,)^27^. Considering that we have only included patients after their first stroke, our baseline creatinine values were lower than other studies that included patients with previous strokes and with higher baseline creatinine values. This might indicate a higher prevalence of previous chronic kidney disease in those studies^9^^,^^26^.

This study had some limitations. Firstly, several patients were excluded from the initial sample due to incomplete data. Although the excluded patients had similar values with respect to age and severity of stroke, a selection bias should not be ruled out. Secondly, our study population represented the reality of a single hospital that is reference center for stroke and dependent of the public health system with certain limitations in intensive care unit. As in other studies, we did not use urinary volume as an additional criterion to AKI definition^28^^,^^29^. Even so, this is the first Brazilian study based on a stroke database with well-defined criteria for the diagnosis of a cerebral event and a current AKI definition used in other epidemiology studies^3^^,^^5^.

## Conclusion

Despite the limitations, our study concluded that AKI is an important complication following a first-ever ischemic stroke and might be an independent predictor of mortality in 30 days when stroke severity is not considered in the analysis.
