# Erratum

**DOI:** 10.1590/2175-8239-JBN-2018-0215er

**Published:** 2019-08-01

In the article “The impact of acute kidney injury on fatality of ischemic stroke from a hospital-based population in Joinville, Brazil”, with DOI code number http://dx.doi.org/10.1590/2175-8239-jbn-2018-0215, published in the Brazilian Journal of Nephrology, Epub ahead of print on May 09, 2019:

The data in Table 1 was originally:

**Table 1:** Baseline characteristics of ischemic stroke in the total sample and by presence or absence of acute kidney injury (AKI)

Total Sample (n = 194)	With AKI (n = 214)	Without AKI (n = 20)	*p* value

The table has been corrected and the numbers should be:

**Table 1:** Baseline characteristics of ischemic stroke in the total sample and by presence or absence of acute kidney injury (AKI)

Total Sample (n = 214)	With AKI (n = 20)	Without AKI (n = 194)	*p* value

